# Automating prostate volume acquisition using abdominal ultrasound scans for prostate-specific antigen density calculations

**DOI:** 10.1038/s41598-025-10420-4

**Published:** 2025-09-30

**Authors:** Rory Douglas Bennett, Tristan Barrett, Nikita Sushentsev, Nimalan Sanmugalingam, Kang-Lung Lee, Vincent J. Gnanapragasam, Zion Tsz Ho Tse

**Affiliations:** 1 School of Engineering and Materials Science, Queen Mary University of London, Mile End Road, London, E1 4NS UK; 2 Department of Radiology, University of Cambridge School of Clinical Medicine, Cambridge, CB2 0QQ UK

**Keywords:** Prostate cancer, Prostate, Biomedical engineering

## Abstract

Proposed methods for prostate cancer screening are currently prohibitively expensive (due to the high costs of imaging equipment such as magnetic resonance imaging and traditional ultrasound systems), inadequate in their detection rates, require highly trained specialists, and/or are invasive, resulting in patient discomfort. These limitations make population-wide screening for prostate cancer challenging. Machine learning techniques applied to abdominal ultrasound scanning may help alleviate some of these disadvantages. Abdominal ultrasound scans are comparatively low cost and exhibit minimal patient discomfort, and machine learning can be applied to mitigate against the high operator-dependent variability of ultrasound scanning. In this study, a state-of-the-art machine learning model was compared to an expert radiologist and trainee radiologist registrars of varying experience when estimating prostate volume from abdominal ultrasound images, a crucial step in detecting prostate cancer using prostate-specific antigen density. The machine learning model calculated prostatic volume by marking out dimensions of the prolate ellipsoid formula from two orthogonal images of the prostate acquired with abdominal ultrasound scans (which could be conducted by operators with minimal experience in a primary care setting). While both the algorithm and the registrars showed high correlation with the expert ($r\ge 0.8$) it was found that the model outperformed the trainees in both accuracy (lowest average volume error of $15\%$) and consistency (lowest IQR of $19\%$ and lowest average volume standard deviation of $13\%$). The results are promising for the future development of an automated prostate cancer screening workflow using machine learning and abdominal ultrasound scans.

**Supplementary Information:**

The online version contains supplementary material available at 10.1038/s41598-025-10420-4.

## Introduction

The prostate gland forms part of the male reproductive system, being located just below the bladder (surrounding the urethra), and produces seminal fluid amongst other substances, including prostate specific antigen (PSA). A “normal”, or healthy, prostate is approximately $3\times 3\times 5\,\text{cm}$ in size, or $25\,{\text{cm}}^{3}$ in volume^1^. Often this size increases with age—known as benign prostatic hyperplasia/enlargement (BPH/E)—which can lead to lower urinary tract symptoms (LUTS) such as urinary hesitancy or abnormally frequent urination and, in some cases, urinary retention^2^. While BPH is non-cancerous, it may require medical intervention if the symptoms affect the patient’s quality of life.

Prostate cancer (PCa) will affect an estimated $13\%$ of men during their lifetime in the United Kingdom (UK)^3^. It is the most diagnosed form of cancer in men within the UK and together with lung, bowel, and breast cancer accounted for nearly half of all cancer related deaths in the UK for the period 2017–2019^4^. PCa has a 5-year survival rate that drops from nearly $100\%$ to $29\%$ in those diagnosed with stage 3 and stage 4 cancer, respectively^5^, highlighting the importance of earlier detection.

A needle biopsy is required to confirm the presence of cancerous tissues in the prostate. There are, however, a variety of pre-biopsy tests that can be performed to determine whether a biopsy is necessary. These tests can help reduce the number of biopsy events and any associated side effects. Digital rectal exams, while low cost and simple to perform, are generally considered inaccurate when determining prostate volume (PV)^6^. Despite this they may be useful when attempting to palpate irregularities in/on the prostate secondary to cancer^7^. Imaging techniques including magnetic resonance imaging (MRI) and transrectal ultrasound (TRUS) can be used to visualise the prostate to more accurately measure PV and MRI has the added ability to detect features of PCa^8,9^. An increase in PSA levels can also result from the presence of PCa. PSA can be detected from a relatively simple blood test and raised levels may indicate PCa. Unfortunately, PSA is not only produced by the prostate^10^**,** and PSA blood levels can increase due to non-cancerous conditions, including BPH^11^. PSA-density (PSAD), which normalises the PSA levels against PV, has been shown to be more reliable than PSA alone when detecting PCa in patients with a Gleason Score $\ge 7$^12^ and can help distinguish between PCa and BPH^13^. Acquiring PV for use in PSAD calculations is an active area of research and is the focus of this study.

There are two main methods that can be used when attempting to estimate PV for PSAD calculations: Stepwise planimetry (full prostate segmentation) and geometric models. Both methods require that the prostate be imaged, with a series of images required for planimetry and (typically) two orthogonal images required for geometric models. Planimetry stacks a series of images together where the prostate boundary has been marked in each image and is the more accurate of the two methods as it can account for irregular boundaries. Geometric models operate under the assumption that the prostate is roughly ellipsoidal in its shape (see Fig. 2b), requiring only three-dimensional measurements to estimate the volume. See Eq. (1), where $L$, $W$, and $H$ are the three major dimensions of the prostate and $C$ is a constant. The value of $C$ is commonly taken as $\frac{\pi }{6}\approx 0.52$ – the prolate ellipsoid assumption. While the geometric model is less accurate than planimetry, it is considered accurate enough^14,15^ and is the PV estimation method used in this study.

1 $$PV=L\times W\times H\times C$$

**Fig. 1:**
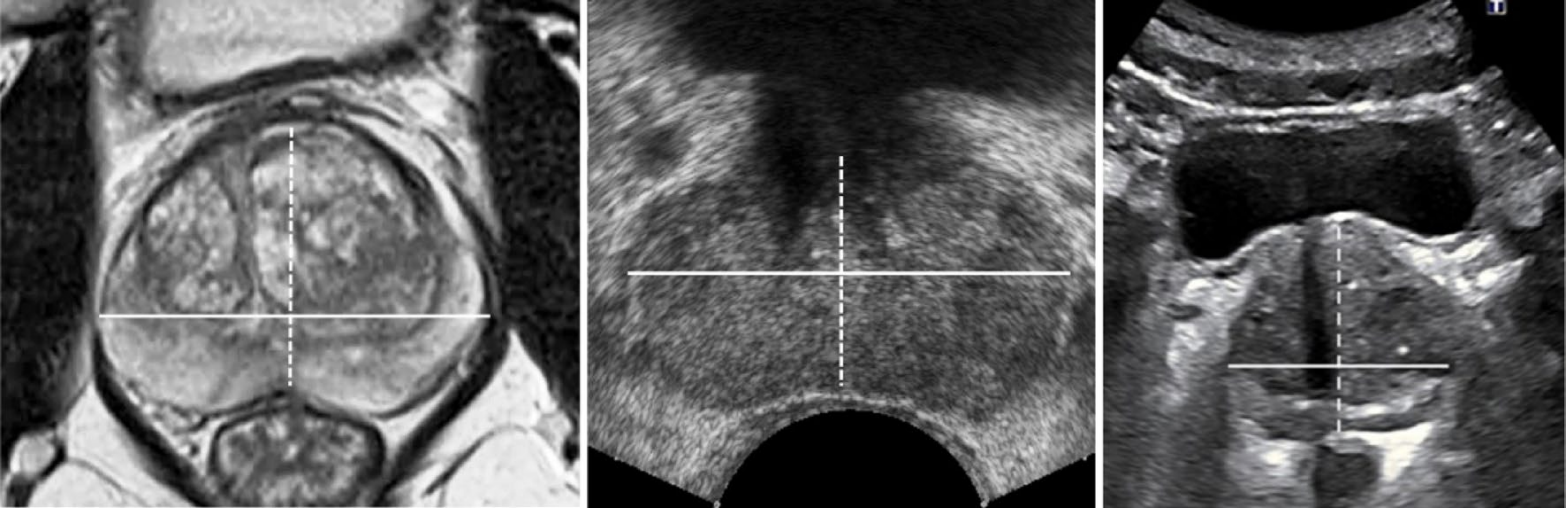
Comparison of different prostate imaging techniques: MRI—Left; TRUS^21^—Middle; and AUS^19^—Right. The major dimensions of the prostate are indicated.

When imaging the prostate for PV estimation either MRI or TRUS is currently used in clinical practice in favour of abdominal ultrasound (AUS). Figure 1 serves to highlight why this is the case. MRI scans of the prostate (left) show a much clearer delineation between the prostate and the surrounding tissues, making prostate boundary detection easier, and subsequent PV estimation more accurate—in comparison to AUS scans (right). TRUS scans of the prostate (middle) show no confounding anatomical structures and have a higher signal-to-noise ratio than AUS scans. However, AUS scans exhibit almost no patient discomfort and are relatively cost effective and easy to perform in comparison to MRI scans and TRUS scans. AUS-derived PV estimates have been shown to have a high correlation with more accurate techniques^16–18^while PSAD values derived from AUS performed by highly experienced operators have been shown to be as effective as MRI-derived PSAD values^19^ and TRUS-derived PSAD values^20^.

**Fig. 2:**
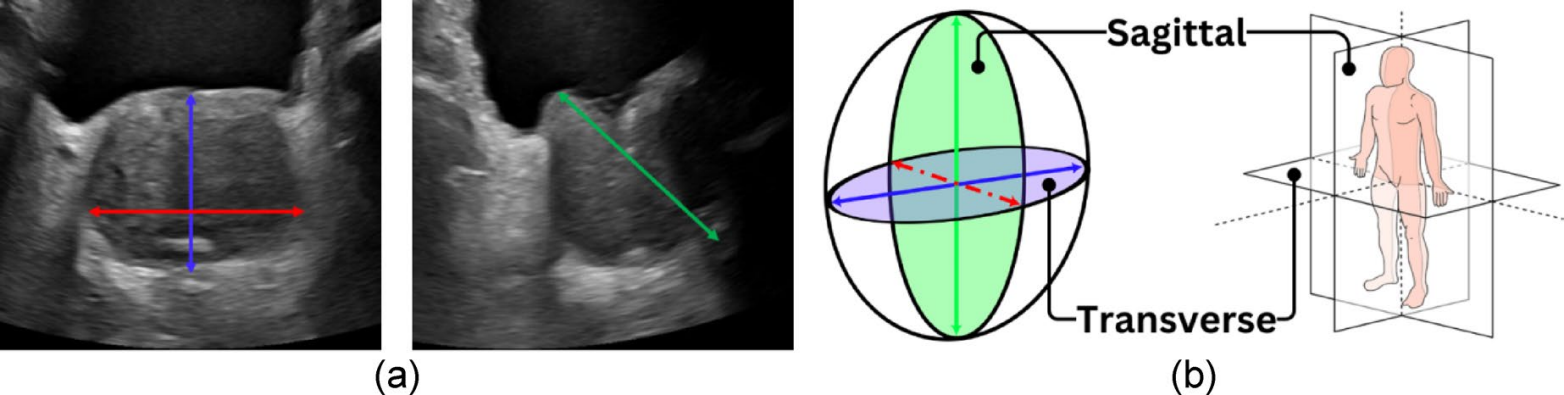
(**a**)—RL (red), AP (blue), and SI (green) dimensions on a tAUS (Left) and sAUS (Right) image. (**b**)—prolate ellipsoid with anatomical planes referenced in this study.

Figure 2 shows how Eq. (1) is used with AUS scans to estimate PV. The locations of the dimensions of Eq. 1 and their relative locations on the anatomical planes are shown. $L$, $W$, and $H$ of Eq. (1) are replaced with right-left (RL—red line), anterior–posterior (AP—blue line), and superior-inferior (SI—green line). The RL and AP dimensions are calculated on a transverse AUS (tAUS) image, while the SI dimension is calculated on a sagittal AUS (sAUS) image.

There is a paucity of research in automated/semi-automated PV estimation using AUS scans, with three studies having published work attempting to automate at least a portion of the PV estimating pipeline using AUS scans of the prostate^22–24^. Betrouni et al.^24^ created an algorithm that segmented a single AUS image of the prostate, taken at roughly the centre of the prostate. While the volume could not be calculated using this method alone, it represented a step towards full prostate segmentation using AUS scans. The signal-to-noise ratios and contrast-to-noise ratios of the images were increased using a contour enhancing filter, while a heuristic optimisation algorithm was used to search for the contour. They found that their system could segment the prostate with a position error of less than $2\,\text{ mm}$. Albayrak et al.^23^ made use of a multi-task deep convolutional neural network (DCNN) to estimate two of the three dimensions of Eq. (1) from tAUS images of the prostate. This DCNN system was trained on multi-scale image patches generated from the tAUS images. The patch creation process helped address the limited dataset size—a known issue with machine learning in general. Again, as with^24^, PV could not be inferred from the output of this DCNN as one dimension was still missing. This was later addressed by the same group when they expanded their DCNN system to infer the last dimension of Eq. (1) from sAUS images of the prostate^22^. When the PVs calculated by this expanded system were compared with volumes calculated by experts, there was a reported mean absolute value difference (MAVD) between their system and expert estimates of $4.95\,\text{ c}{\text{m}}^{3}$ (the error metric MAVD is expanded on in the Methods section). This expanded system forms the basis of this study and was tested on a newly acquired clinical trial patient dataset (see Methods section).

## Methods

### Datasets

#### Original dataset

The original dataset as reported in^22^ was made up of 305 patients. One central transverse image and one central sagittal image of the prostate was acquired from each patient, with expert markings given. No patient demographics were given.

#### Clinical patient dataset

A new clinical patient dataset was acquired prospectively as part of this study. 19 patients who had been referred for suspected PCa were included. 18 patients were under active surveillance with one patient pre-biopsy. All patients signed written, informed consent (NRES Committee East of England, UK, ref: NRES 03/018). The median patient age was 75 years, ranging from 60 to 82 years old. The patients had undergone an MRI scan of the prostate within six months of them having the ultrasound scan which was used to calculate the MRI-derived PVs for each patient.

Patients were asked to ensure that they had a full bladder before their AUS scanning session. An experienced uro-radiologist conducted eight sweep scans per patient: four transabdominal scans (comprising of two transverse and two sagittal sweeps) and four transperineal scans (comprising of two transverse and two sagittal sweeps). A Canon Aplio i700 ultrasound system using a curved linear array US transducer with a centre frequency of $3.5\,\text{ MHz}$ and a range of $1.9\,\text{ MHz}$—$6\,\text{ MHz}$ was used. The scanned images were immediately synced with orientation data on a laptop that was streaming both the images from the ultrasound system and the orientation data. Only the transabdominal scans were used in this study. The orientation data was streamed from the commercially available Witmotion BWT901CL Bluetooth 9-axis sensor.

### Model

The model presented in^22^ was used in this study; a brief overview of its operating principles is given below. The model makes use of a patch-based voting system and is referred to as the image-patch voting (IPV) model. The patches that are generated serve to substantially increase the training and inference dataset size. A known limitation of typical patch-based models is that they can only extract local information due to the patches being markedly smaller than the original image. To address this, patches of four different sizes are created to allow for more generalised information extraction, without losing localised details. The four patches have the following square dimensions: 64, 128, 256, and 512 pixels. Each patch is then down sampled to 64-by-64 pixels. The network is called a quadruplet DCNN (QDCNN) as it makes use of four ResNet-18 DCNNs. Each ResNet-18 DCNN is trained on one of the four patches.

#### Model operation

**Fig. 3:**
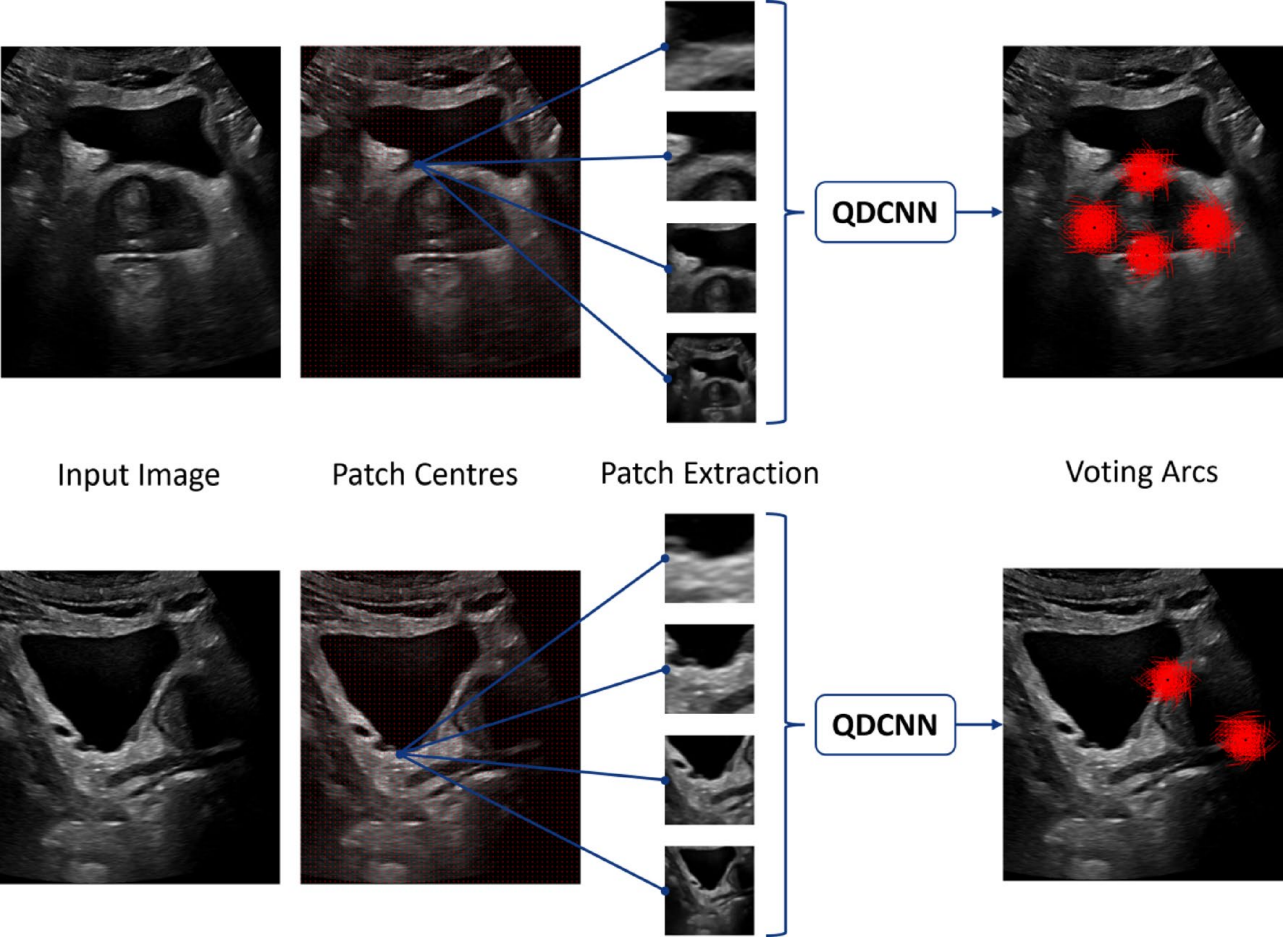
IPV inference workflow^22^, from initial image to patch extraction to voting arcs used for inference. Top—A sample tAUS image workflow. Bottom—A sample sAUS image workflow. QDCNN represents either the transverse model, or the sagittal model.

The IPV model attempts to locate six points, corresponding to the endpoints of the three dimensions of Eq. (1), from two orthogonal AUS scans of the prostate (one transverse image, one sagittal image). Four points are located on the tAUS image (RL and AP dimension), whereas two points are located on the sAUS image (SI dimension). This requires that there are two independently trained systems: one system is trained to infer four points on tAUS images (transverse model), while the second system is trained to infer two points on sAUS images (sagittal model). Four points are located on the tAUS image as better visualisation of the prostate boundary is possible. The steps below give an overview of the models’ operation during inference:

1. Receive scaled ($40\frac{pixels}{\text{cm}}$) input image. Either a tAUS image or a sAUS image. Scaling is used for comparative reasons; it is not necessary for the model to function.
2. Extract patches. Patch centre points are uniformly distributed across the input image. From each centre point four new patches of increasing size are generated. These patches are then down sampled to 64-by-64 pixels.
3. Pass extracted patches through relevant model. Each patch results in voting arcs, indicating where dimension end points are in relation to the centre of the patch.
4. Combine all votes from all patches. This results in areas where the endpoints are most likely to be located. Figure 3 shows a sample of the generated voting arcs for both a tAUS image and a sAUS image. The density of the voting arcs can be thought of as the models’ confidence in the endpoint locations.

$k$-fold cross-validation was employed in the original study ($k=10$) to understand how well the IPV model performed on average, with the same approach ($k=10$) being followed in the present study. $k$-fold cross-validation splits the original dataset into $k$ different training and validating subsets, or folds, resulting in trained models with differing final parameters as different training images are used.

Two sets of models were trained: Original and augmented. The original models were trained only on the original dataset, following the same fold structure. The augmented models were trained using a combination of the original dataset and 9 sets (sagittal and transverse images from matching patients) from the newly acquired patient dataset.

### PV markers

For this study five PV estimates (including individual dimensions) measured by one expert uro-radiologist and three trainee radiologist registrars were compared (see Table 1). First, the expert AUS measurements were compared with the gold standard MRI measurements, with MRI assumed to be more accurate in accordance with literature^14,15^. Next, a comparison between the original and augmented models’ measurements, using the expert AUS measurements as reference, was carried out. Finally, the augmented models’ and trainee radiologist registrars’ measurements were compared using the expert AUS measurements as reference.

**Table 1:** Description of the five groups of markers that located the dimensions of Eq. (1) on either MRI scans or AUS scans of the prostate.

Marker	Description
Expert MRI	An expert uro-radiologist, the same radiologist who conducted the patient scans, marked the dimensions of Eq. (1) on the MRI scans of all 19 patients. The dimension values were then reported. The middle slice of the prostate was used to mark the RL and AP dimensions, whereas the SI dimension was taken as the distance between the first and last slices that contained images of the prostate
Expert AUS	The same expert marked the dimensions of Eq. (1) on the central tAUS and sAUS images of all 19 patients
Trainee radiologist registrars	Three trainee registrars with varying degrees of experience in marking AUS scans of the prostate were asked to locate the dimensions of Eq. 1 on the central tAUS and sAUS images of all 19 patients. They were presented with the same image as chosen by the expert radiologist
Original IPV model	These models were trained solely on the dataset made available by the original study, following the same fold structure. All 19 patients’ images were then passed to the model for inference
Augmented IPV model	A portion of the newly acquired dataset was added to the original dataset used during training to increase the models’ robustness when dealing with a more varied dataset. All 19 patients’ images were then passed to the model for inference

### Performance metrics

Various performance metrics can be used to analyse the efficacy of a machine learning model. The error metric used in the original study was the MAVD value, as defined in Eq. (2), where $N$ is the number of samples, ${M}_{expert}$ and ${M}_{x}$ are two different markers (expert marker and IPV model/registrar respectively), and ${V}_{expert}$ and ${V}_{x}$ are the two volumes calculated from the dimensions of the two markers using Eq. (1). The MAVD can be thought of as the average difference between two markers across the dataset. For the IPV models’ measurements the average dimension value across the 10 folds was used in the volume MAVD calculation, ignoring failed measurements. For individual dimensions (RL, AP, SI) a failed measurement occurred when at least one end point was not inferred by the model. If any of the dimension measurements failed, so would the related volume measurement.

2 $$MAVD\left({M}_{expert}, {M}_{x}\right)=\frac{1}{N}\sum_{j=1}^{N}\left|{V}_{expert}^{j}-{V}_{x}^{j}\right|$$

Reporting MAVD alone can be misleading. The performance of a model with a resultant MAVD of $5\,\text{ c}{\text{m}}^{3}$ varies greatly depending on the true/reference value. If the reference value is $80\,\text{ c}{\text{m}}^{3}$ then the MAVD value of $5\,\text{ c}{\text{m}}^{3}$ represents an acceptable error of 6.25 $\%$. If, however, the reference value is closer to $25\,\text{ c}{\text{m}}^{3}$ then the MAVD of $5\,\text{ c}{\text{m}}^{3}$ represents a less acceptable error of $20 \%$. For this reason, both the MAVD as presented above and the MAVD as a percentage of the true value are reported. The calculation of MAVD as a percentage of the true value (MAVD*) is shown in Eq. (3).

3 $$MAV{D}^{*}\left({M}_{expert}, {M}_{x}\right)=\frac{1}{N}\sum_{j=1}^{N}\frac{\left|{V}_{expert}^{j}-{V}_{x}^{j}\right|}{{V}_{expert}^{j}}\times 100$$

While the original study only considered the MAVD of the final calculated PVs between the IPV model and experts it was decided to include a comparison of the individual dimensions of Eq. (1) in this study. ${V}_{expert}$ and ${V}_{x}$ of Eq. (2) were replaced with individual corresponding dimensions. The standard deviation of the absolute value difference (SDAVD) and the standard deviation of the absolute value difference as a percentage of the true value (SDAVD*) were calculated as the standard deviations of both the MAVD and MAVD* values, respectively.

Correlations between the IPV results (original and augmented) and the expert values, and between the registrars results and the expert values, were calculated using Pearson’s correlation coefficients (PCCs). The average values (dimensions and volumes) returned by the models over the 10 folds were used for PCC calculations. A correlation coefficient of $0.7$ was taken as representing a high correlation^25^.

To show the agreement between the various techniques Bland–Altman (difference) plots^26^ were generated showing the limits of agreement as $\overline{d }\pm 1.96s$, where $\overline{d }$ is the mean difference and $s$ the standard deviation of the differences. Bland–Altman plots were generated to compare the expert MRI derived volumes with the expert ultrasound derived volumes, and the expert ultrasound derived volumes with the IPV models’ (original and augmented) derived volumes.

Statistical analyses were conducted when comparing the models to the trainee radiologist registrars. First, Friedman tests were carried out to determine if there were statistically significant differences between the groups (dimension values and MAVD* values). This was followed by Wilcoxon sign-ranked tests (dimension values and MAVD* values). For the PCCs, Fisher’s $r$-to-$z$ transformation was used for comparisons.

## Results

### MRI vs AUS

**Fig. 4:**
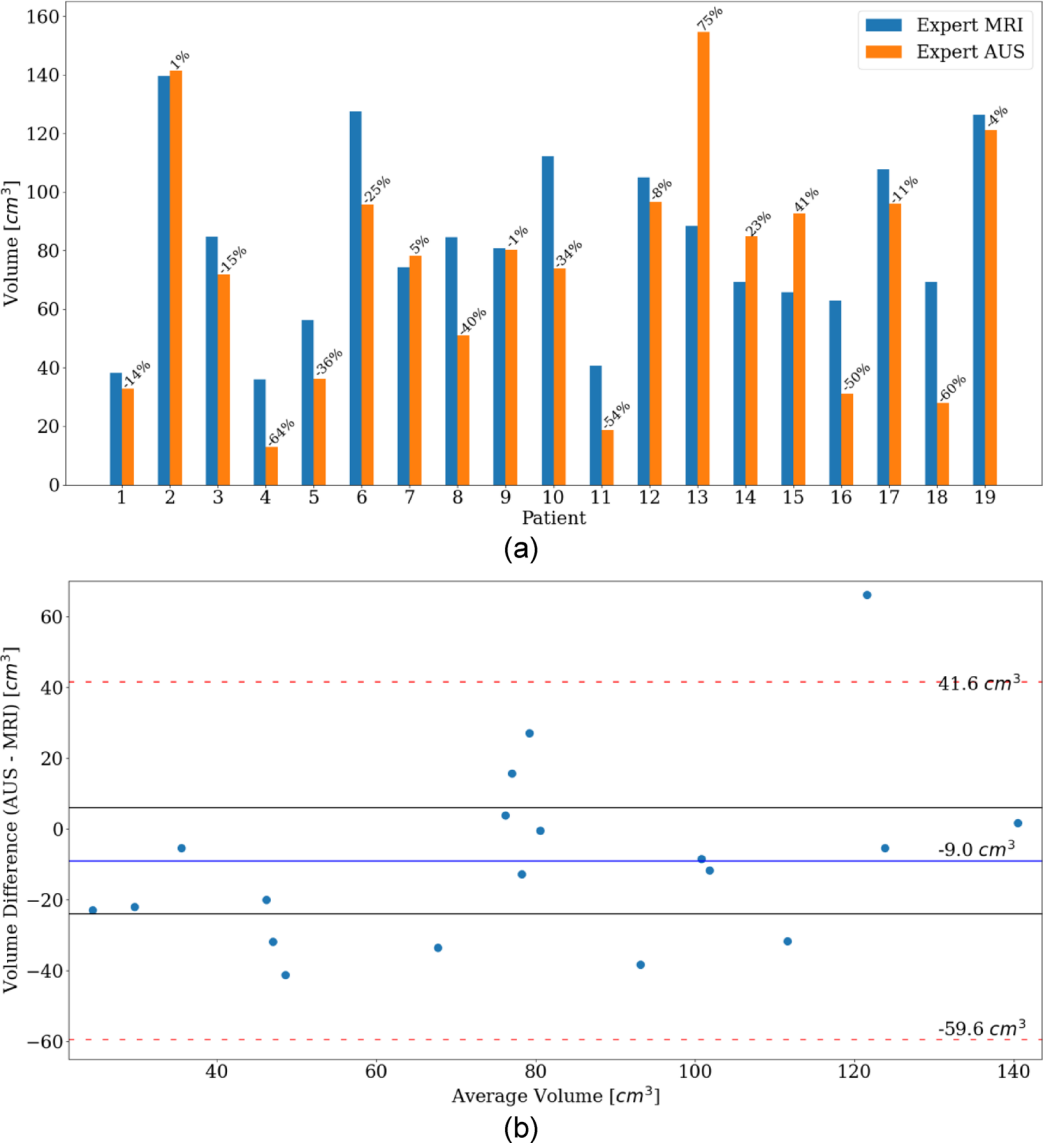
(**a**) MRI and AUS estimated PV for each patient with percentage difference given. (**b**) Bland Altman analysis plot of PV measured by MRI and AUS. $y$ axis indicates volume difference. $x$ axis indicates the average volume between the two measurements. LOAs shown with red dashed line. Mean difference shown with blue line. Black lines indicate $\pm 15\,\text{ c}{\text{m}}^{3}$ of mean difference.

Expert MRI volumes and expert AUS volumes for each patient are shown in Fig. 4a. The larger prostate sizes and the high variability were expected due to the age of the participants. For the Bland-Altman plot in Fig. 4b the mean difference value was $-9\,\text{ c}{\text{m}}^{3}$, with LOAs of $-59.6\,\text{ c}{\text{m}}^{3}$ and $41.6\,\text{ c}{\text{m}}^{3}$. Expert AUS volumes showed underestimation in 14 cases and overestimation in 5 cases, with 1 case falling outside the LOAs. 11 cases were shown to have a difference that fell within $\pm 15\,\text{ c}{\text{m}}^{3}$ of the mean difference.

### IPV models and registrars

First, the original models’ and augmented models’ measurements were compared taking the expert AUS measurements as reference. Second, the three registrars’ measurements were compared with the augmented models’ measurements and the expert AUS measurements.

#### Original IPV model(s) versus augmented model(s)

**Fig. 5:**
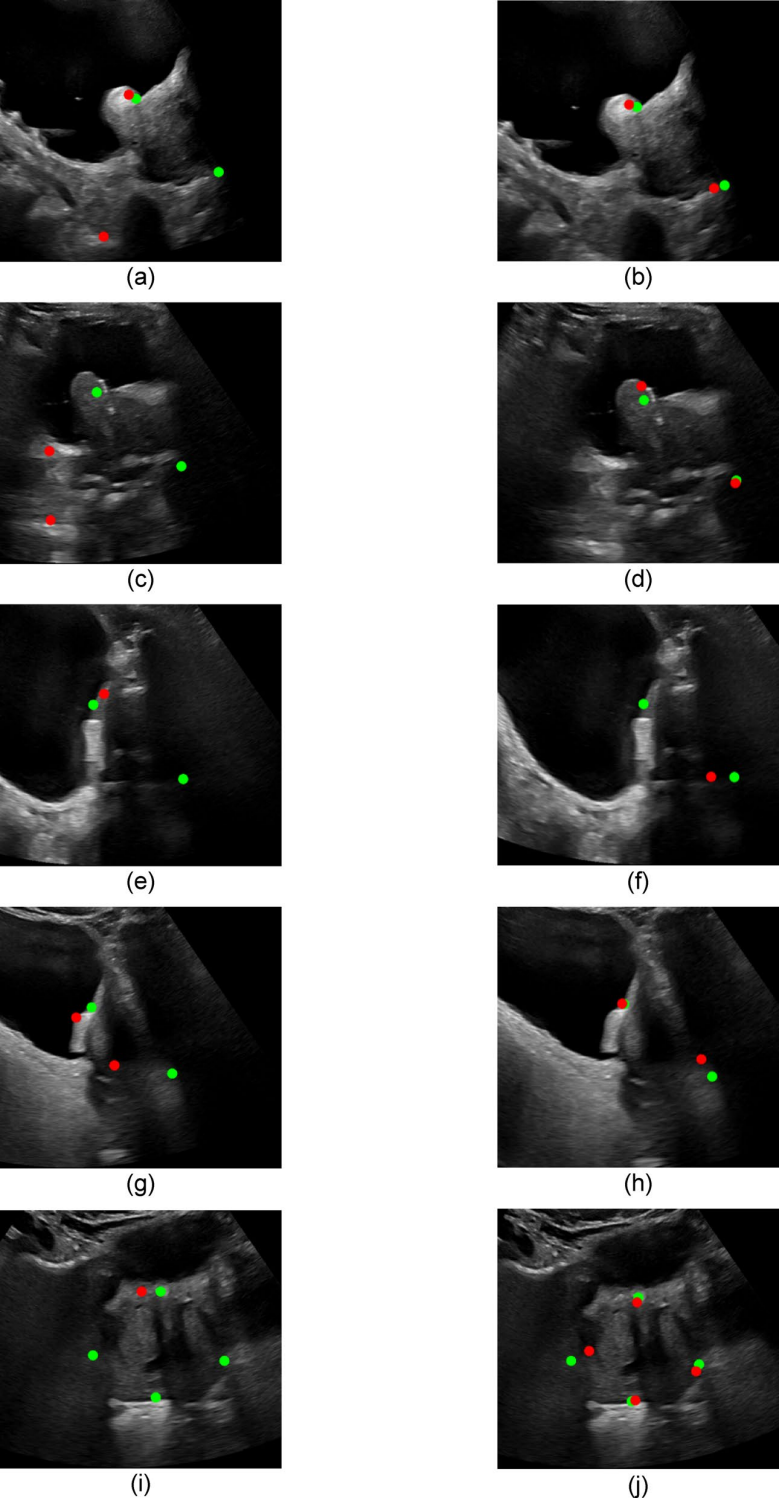
Sample original models’ results (left column) compared with augmented models’ results (right column). Green points indicate expert AUS markings; red points indicate model inferred markings. (**a**–**h**) show sagittal results (SI measurement) while (**i**,**j**) show transverse results (RL and AP measurements).

Figure 5 shows sample results returned by the original models (left column) and how the augmented models (right column) performed on the same images. (a) through (h) are sAUS images, whereas (i) and (j) are tAUS images. The original models performed exceptionally well in identifying the tAUS endpoints except in the case which is shown in (i), where three of the four endpoint locations were not identified at all. The result shown is indicative of how the original model performed across multiple folds for this specific image. (a), (c), and (e) show examples of where the original models incorrectly identified one or more of the SI dimension’s endpoints, while (g) shows substantial underestimation of the SI dimension. (b), (d), and (h) show a substantial improvement by the augmented models in comparison to corresponding results of the original models. The only case that did not show improvement is shown in (e) and (f), where the original and augmented models both failed to identify an endpoint, making calculation of the respective SI dimension, and resultant volume, impossible. (f) and (h) show examples of correct identification of the distal end of the prostate, even though it is obscured by pubic bone shadow.

**Fig. 6:**
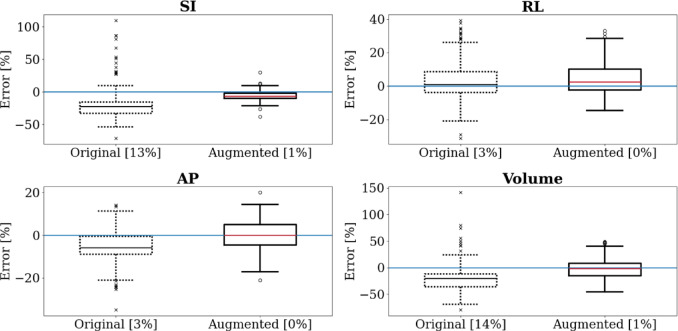
Box and whisker plots showing original and augmented models’ performance on the new dataset for all measurements. Whiskers indicate $1.5\times IQR$. Medians shown in black and red. Outliers shown with crosses and circles. Errors (y-axes) are expressed as a percentage of expert AUS values taken across all folds. Values in parentheses on the $x$-axes indicate how often the models failed to infer a measurement as a percentage [original/augmented]. Blue line indicates zero error.

**Fig. 7:**
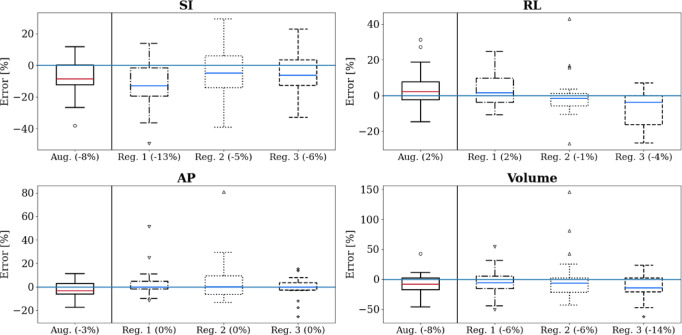
Box and whisker plots showing augmented model(s)’ and registrars’ performance on the new dataset for individual dimension measurements and resultant volume measurements. Whiskers indicate $1.5\times IQR$. Medians shown in red (model), and blue (registrars). Outliers shown with circles (augmented model(s)), down triangles (registrar 1), up triangles (registrar 2), and stars (registrar 3). Errors (y-axes) are expressed as a percentage of expert AUS values. Median error is given in parentheses. Blue line indicates zero error.

Figure 6 is a box and whisker plot summarising the performance of the original and augmented models across the 10 folds for each inferred measurement. These inferred measurements were compared to expert AUS measurements. Corresponding numerical values—mean, IQR, and median—can be found in Supplemental Materials Table 1, Table 2, and Table 3, respectively. Unlike Fig. 7, the measurements in Fig. 6 were not averaged over the folds. This approach provides a clearer picture of model consistency pre- and post-augmentation. A tighter box indicates more consistency across folds versus a more spread-out box. A measurement was considered failed if at least one endpoint of a dimension could not be inferred. Volume estimation was considered failed if any of its three dimensions failed.

**Table 2:** MAVD values ($\text{mm}$ or $\text{ml}$) and MAVD* ($\text{\%}$) for the results of the two models and four registrars when compared with the expert AUS measurements. Bold values indicate lowest MAVD* values.

Marker	SI		RL		AP		Volume	
	$\text{mm}$	$\text{\%}$	$\text{mm}$	$\text{\%}$	$\text{mm}$	$\text{\%}$	${\text{cm}}^{3}$	$\text{\%}$
Original	11.75	23.15	4.04	8.49	3.47	7.19	19.56	26.91
Augmented	5.25	10.8	4.04	8.54	2.91	6.44	9.25	14.98
Registrar 1	7.8	15.53	4.08	8.1	3.22	7.63	10.54	17.88
Registrar 2	7.35	13.91	4.02	8.51	5.05	12.25	16.46	27.23
Registrar 3	6.45	12.21	5.29	9.98	3.1	6.31	14.17	19.9

**Table 3:** PCCs for the two models and three registrars when compared with the expert AUS measurements. Bold values indicate largest correlations in group.

Marker	SI	RL	AP	Volume
Original ($p<.001$)	$0.74$	$0.85$	$0.96$	$0.92$
Augmented ($p<.001$)	$0.93$	$0.86$	$0.96$	$0.97$
Registrar 1 ($p<.001$)	$0.84$	$0.9$	$0.88$	$0.94$
Registrar 2 ($p<.01$)	$0.77$	$0.84$	$0.76$	$0.79$
Registrar 3 ($p<.001$)	$0.86$	$0.87$	$0.9$	$0.91$

Considering the SI measurements, the augmented model outperformed the original, showing a smaller median error and IQR (see Supplemental Materials), fewer outliers and failures, and reduced underestimation. For the RL measurement, the key improvements were fewer outliers and failures, although the median errors and IQRs remained similar. For the AP measurement, the augmented model had fewer outliers and failures, a lower median error, and a similar IQR to the original. Finally, in the volume measurements, the augmented model showed improvements in median error, number of outliers, and number of failures. Both models exhibited similar IQRs and tended towards underestimation of the volume, though this underestimation was less pronounced in the augmented model.

#### Registrars’ markings

Figure 7 summarises the performance of the registrars along with the augmented models across each measured dimension and resulting volume measurement. The values used for the models’ dimensions were averaged over the folds, with the model volumes calculated using the same averaged dimensions. This gives a better idea of how the models performed on average, as opposed to the model consistency comparisons of Fig. 6. The failed dimension was excluded from the augmented models’ results. Corresponding numerical values—mean, IQR, and median—can be found in Supplemental Materials Table 1, Table 2, and Table 3, respectively.

For the SI measurement: registrar 2 showed the lowest mean and median, whereas the augmented models showed the lowest IQR. For the RL measurement: registrar 2 showed the lowest mean, IQR, and median. For the AP measurement: registrar 3 showed the lowest mean and IQR, and registrar 1 showed the lowest median. For the volume measurement: registrar 2 showed the lowest mean, the augmented models showed the lowest IQR, and registrar 1 the lowest median. Friedman and Wilcoxon tests were conducted on the absolute percentage errors across measurements. While the augmented model showed some improvements over the original model, across all dimensions, the augmented model’s performance was statistically the same as the trainee radiologist registrars.

### Performance metrics

#### MAVD and MAVD* values

Calculated MAVD and MAVD* values for each marker (original and augmented models and three registrars) are shown in Table 2, with corresponding SDAVD and SDAVD* values given in Supplemental Materials Table 4. Failed model results were excluded from calculations.

Registrars 1 and 2 had the fewest number of errors when estimating the RL and AP measurements, respectively, whereas the augmented models showed better performance (lower MAVD*) when estimating the SI dimension, and more importantly, when estimating the volume. Friedman and Wilcoxon tests for the SI and volume measurements showed that the augmented model significantly outperformed the original model ($p<0.001$ and $p=0.001$, respectively). No statistically significant differences were found for the RL or AP measurements ($p>0.05$ in both Friedman tests). These results indicate that model augmentation led to substantial improvements in MAVD* for SI and volume measurements, while maintaining performance comparable to trainee radiologist registrars across all dimensions.

#### Pearson’s correlation coefficients

The PCCs are presented in Table 3, where failed model results were excluded from calculations. Significantly strong correlation was found across all measurements for both the original and augmented models when compared with the expert AUS measurements, with the lowest correlation shown for the original models’ SI measurement. This was improved in the augmented models results. The only cases when a registrar showed a higher correlation than the augmented models was registrar 1 and registrar 3 when estimating the RL measurement, although this difference was only marginal. The PCCs were then compared using Fisher’s $r$-to-$z$ transformation. The augmented model had a significantly stronger correlation than the original model to expert AUS markings for the SI measurement ($z=1.97$, $p=0.049$), while no significant differences were found in the RL dimension across evaluators ($p>0.6$ for all comparisons). Comparisons between the augmented model and trainee radiologist registrars in the SI, AP, and the volume measurements showed moderate to large $z$-scores, however, they did not reach statistical significance ($p>0.05$). This was likely due to the limited size of the dataset.

#### Bland-Altman plots

**Fig. 8:**
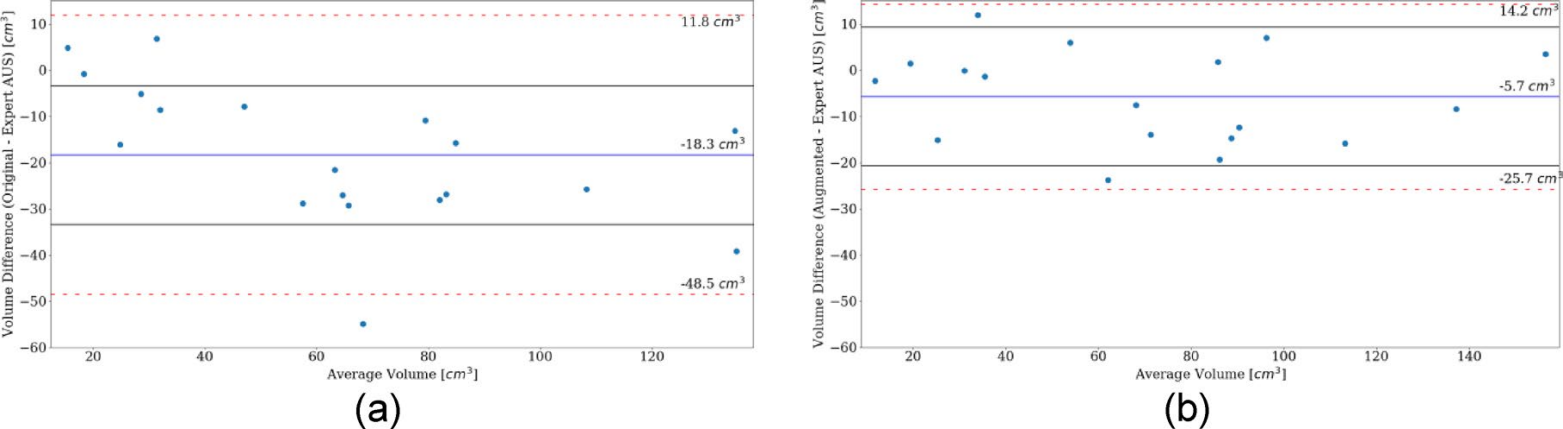
Bland Altman plots showing the agreement between expert AUS volumes and original models’ volumes (**a**) and expert AUS volumes and augmented models’ volumes (**b**). $y$ axes indicate volume difference. $x$ axes indicate the average volume between the two measurements.

Comparing expert AUS markings and original models’ markings (Fig. 8a): The mean difference value was $-18.3 \,{\text{cm}}^{3}$, with LOAs of $-48.5\text{ c}{\text{m}}^{3}$ and $11.8\text{ c}{\text{m}}^{3}$. 17 original models’ volumes underestimated their respective expert AUS values, 2 overestimated, 1 difference fell outside the LOAs, with 14 points falling within $\pm 15\text{ c}{\text{m}}^{3}$ of the mean difference. Expert AUS markings and augmented models’ markings (Fig. 8b): The mean difference value was $-5.7\text{ c}{\text{m}}^{3}$, with LOAs of $-25.7\text{ c}{\text{m}}^{3}$ and $14.2\text{ c}{\text{m}}^{3}$. 12 augmented models’ volumes underestimated their respective expert AUS values, 6 overestimated, no differences fell outside the LOAs, with 16 points falling within $\pm 15\text{ c}{\text{m}}^{3}$ of the mean difference. One patient’s volume calculation was not included in Fig. 8b as the augmented models failed to calculate the SI dimension.

## Discussion

Expert MRI and expert AUS markings were found to have a high correlation with a general lack of agreement. There was a tendency towards improvement in the performance of the augmented models in comparison to the original models with the augmented models outperforming the registrars in PV estimates.

### Expert MRI vs expert AUS

The PCCs showed a high degree of correlation across all dimension measurements and volume measurement, in agreement with previous studies^16–19,27–32^. The Bland-Altman analysis showed that even though the correlation between expert MRI-derived measurements and expert AUS-derived measurements was high, the agreement between the two methods was less so—as evidenced by the large LOAs. This lack of agreement between expert MRI markings and expert AUS markings using the Bland-Altman analysis has been reported previously^33^, however, this does not mean that AUS-derived PVs have no utility, especially in the calculation of PSAD. Pantelidou et al.^19^ showed AUS-derived PSAD values could achieve sensitivities and specificities of $100\%$ in comparison to MRI-derived PSAD values when deciding whether a patient should undergo a biopsy.

### Original vs augmented IPV models

Certain features of the new dataset presented themselves as problematic during inference when using the original models. The augmented models were created to incorporate these new features. The images used to augment the original dataset were specifically chosen to include features that were missing from the original dataset. The demographics of the original dataset were not available, however, the newly acquired patient dataset was made up of older patients. This resulted in prostates that were more irregularly shaped, displaying varying degrees of prostatic protrusion, and some prostates that were obscured by pubic bone shadowing—hiding the distal end of the prostate from view in the sagittal plane (see Fig. 5e,g). 9 transverse and 9 sagittal images (from matching patients) were added to the training pool of the original dataset, representing an increase of only $3\%$ of the original dataset size.

The original models showed poor performance when locating the SI endpoints on the sAUS images. A substantial number of failures occurred, with a considerable number of endpoints being placed in the incorrect location ($7.6\%$ and $9.5\%$ for the SI endpoints, respectively). In an automated system failed endpoints can be detected easily and flagged for further investigation by a clinician, however, incorrectly located endpoints present themselves as a unique challenge. In a worst-case scenario, the incorrectly inferred endpoints pass through unnoticed, resulting in volume estimates that are entirely incorrect. Thus, the improvement in incorrectly inferred endpoints’ locations is considered significant. Any clinical implementation of such a system would need to consider the possibility of incorrectly identified endpoints, with suitable steps in place to identify and deal with them. Even though the original models showed a correlation of $>0.7$ with the expert AUS SI measurements, the MAVD* was still relatively high ($23.15\%$). All SI performance metrics were improved by the augmented models (MAVD* down to $10.8\%$ and PCC up to $0.93$). Only one augmented model case failed (see Fig. 5j), however, it was found that this failure could be rectified by simply altering the fold structure during training, or by increasing the number of patches generated during inference.

The original models performed quite well when locating the RL and AP endpoints on the tAUS images. This was shown by the relatively low number of failures in estimating the RL and AP dimensions ($3\%$ each), with no endpoints inferred in the incorrect location. There was one case that proved difficult for the original models (see Fig. 5i), where 6 of the 10 folds failed to identify one or more endpoints in the image. The augmented models improved on this, showing no failures of the RL and AP dimensions across all 10 folds. The MAVD* and SDAVD* values of the original and augmented models in the RL and AP dimensions were shown to be similar, all below $10\%$. They also showed similar correlation values with expert AUS values, all $>0.8$. Since the original models performed quite well on the transverse images, there was very little improvement to be made. It was for this reason that the extra images added to the original dataset only focused on features present in the sAUS images.

The original models’ volume estimates were improved on by the augmented models, with a drop in the MAVD* values (from roughly $27\%$ to about $15\%$) and failures (from just above $14\%$ to $1\%$), while the correlation was marginally increased (from $0.92$ to $0.97$). The Bland-Altman plots (Fig. 8) showed a tendency of both models to underestimate the expert AUS measurements, while a reduced mean difference, and a reduced LOA range from the augmented models was evident. While this was not surprising as the augmented models where better tuned to handle the new patient dataset it is a good confirmation of improvement in agreement. Although both original and augmented models tended towards underestimation of the expert volumes the augmented models did show a more even spread between underestimation and overestimation.

In comparison to the results presented in the original study^22^, where a final MAVD value of $4.95\text{ c}{\text{m}}^{3}$ for the volume estimates was shown, the MAVD of the of the volume for the augmented models in the present study was $9.25\text{ c}{\text{m}}^{3}$. Unfortunately, since patient demographics were never published in the original paper, it is difficult to make a direct comparison between our results and theirs. However, if we convert their $4.95\text{ c}{\text{m}}^{3}$ to a percentage assuming an average prostate size range of $25\text{ c}{\text{m}}^{3}$ (a healthy prostate) to $50\text{ c}{\text{m}}^{3}$ (used to stratify between smaller and larger prostates^20,32^), the percentage error range would be about $20\%$ to $10\%$, a range where the current error of $15\%$ is central.

### Registrars’ vs augmented IPV models

**Fig. 9:**
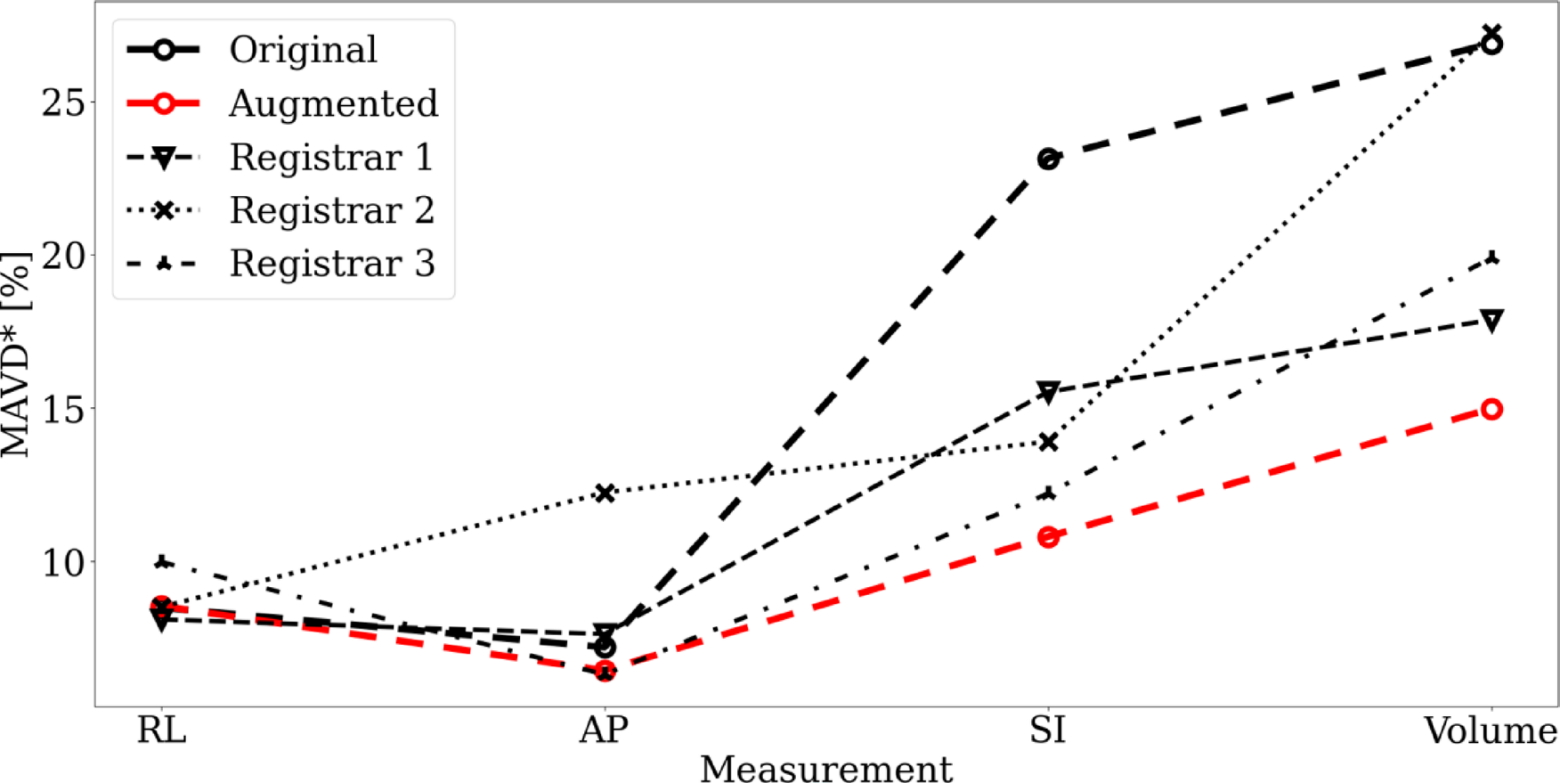
MAVD* values for the original, augmented, and three registrars for all measurements made and resulting PV.

Looking at the registrars’ and augmented models’ results in comparison to the expert AUS measurements they appeared to be very similar, both in MAVD* values and correlation values. In some cases, a registrar outperformed the augmented models, in other cases the augmented models performed better. Taking the MAVD* of the volume to be the deciding factor for accuracy (as volume is used to calculate PSAD) the augmented models outperformed all the registrars, as a group or individually. The augmented models performed substantially better than the original models and two of the registrars in this regard. This is highlighted in Fig. 9, where the performance of the original models, augmented models, and registrars are compared.

Although the individual dimension results do not show a clear distinction between registrar experience, the MAVD* of the volume does, with registrar 1 showing the lowest MAVD* value, followed by registrar 3, and then registrar 2. However, a larger group of registrars marking a larger dataset would be needed to improve the robustness of this (preliminary) analysis comparing the IPV model with trainee radiologist registrars.

## Conclusion

A state-of-the-art machine learning model that can be used to estimate PV from AUS scans of the prostate has been tested on a newly acquired clinical patient dataset. The augmented model was shown to perform about as well as trainee radiologist registrars when estimating the individual dimensions of the prolate ellipsoid equation, and moderately better than the trainee registrars when estimating PV. These results represent a promising step towards automating the calculation of PSAD using AUS scans of the prostate and could potentially lead to development of point-of-care ultrasound systems for use as a triage step in PCa screening.

## Electronic supplementary material

Below is the link to the electronic supplementary material.

Supplementary Material 1

## Data Availability

Data is provided within the manuscript or supplementary information files.
