# Supplementary material for: Automating prostate volume acquisition using abdominal ultrasound scans for prostate-specific antigen density calculations
Source: Sci Rep. 2025 Sep 30;15:33937. doi: 10.1038/s41598-025-10420-4 (PMC12484655; doi:10.1038/s41598-025-10420-4)
Supplement: Supplementary file 1 — Supplementary Material 1 [file 41598_2025_10420_MOESM1_ESM.docx]

# Automating Prostate Volume Acquisition Using Abdominal Ultrasound Scans for Prostate-Specific Antigen Density Calculations

**Rory Douglas Bennett^1^, Tristan Barrett^2^, Nikita Sushentsev^2^, Nimalan Sanmugalingam^2^, Kang-Lung Lee^2^, Vincent J. Gnanapragasam^2^, Zion Tsz Ho Tse^1, *^**

^1^School of Engineering and Materials Science, Queen Mary University of London, Mile End Road, London, UK, E1 4NS.

^2^Department of Radiology, University of Cambridge School of Clinical Medicine, Cambridge, UK, CB2 0QQ.

^*^z.tse@qmul.ac.uk

Table 1: Absolute mean of the percentage error values for the dimension and volume measurements of the augmented model(s) and registrars with respect to expert AUS measurements. Bold values indicate best performing marker in group.

| Marker | SI [%] | RL [%] | AP [%] | Volume [%] |
| --- | --- | --- | --- | --- |
| Augmented | $7.96$ | $3.66$ | $2.28$ | $6.79$ |
| Registrar 1 | $11.94$ | $3.82$ | $4.05$ | $4.53$ |
| Registrar 2 | $\boldsymbol{3.92}$ | $\boldsymbol{0.04}$ | $5.78$ | $\boldsymbol{4.35}$ |
| Registrar 3 | $5.28$ | $7.24$ | $\boldsymbol{0.84}$ | $11.76$ |

Table 2: IQR of the percentage error values for the dimension and volume measurements of the augmented model(s) and registrars with respect to expert AUS measurements. Bold values indicate best performing marker in group.

| Marker | SI [%] | RL [%] | AP [%] | Volume [%] |
| --- | --- | --- | --- | --- |
| Augmented | $\boldsymbol{12.61}$ | $10.04$ | $9.2$ | $\boldsymbol{19.37}$ |
| Registrar 1 | $17.64$ | $13.54$ | $6.47$ | $21.3$ |
| Registrar 2 | $20.19$ | $\boldsymbol{6.88}$ | $15.79$ | $23.9$ |
| Registrar 3 | $16.3$ | $16.25$ | $\boldsymbol{6.1}$ | $23.11$ |

Table 3: Absolute median of the percentage error values for the dimension and volume measurements of the augmented model(s) and registrars with respect to expert AUS measurements. Bold values indicate best performing marker in group.

| Marker | SI [%] | RL [%] | AP [%] | Volume [%] |
| --- | --- | --- | --- | --- |
| Augmented | $8.4$ | $2.11$ | $3.13$ | $8.24$ |
| Registrar 1 | $12.95$ | $1.59$ | $\boldsymbol{0.1}$ | $\boldsymbol{5.92}$ |
| Registrar 2 | $\boldsymbol{4.83}$ | $\boldsymbol{1.44}$ | $0.21$ | $6.08$ |
| Registrar 3 | $6.16$ | $3.76$ | $0.22$ | $14.47$ |

Table 4: SDAVD values ($mm$ or $ml$) and SDAVD* ($\%$) values for the two models and four registrars when compared with the expert AUS measurements. Bold values indicate lowest SDAVD* values.

| Marker | SI | | RL | | AP | | Volume | |
| --- | --- | --- | --- | --- | --- | --- | --- | --- |
|  | $mm$ | $\%$ | $mm$ | $\%$ | $mm$ | $\%$ | $ml$ | $\%$ |
| Original | $5.96$ | $12.6$ | $3.48$ | $8.27$ | $2.9$ | $5.24$ | $13.33$ | $13.11$ |
| Augmented | $3.71$ | $8.82$ | $3.42$ | $8.69$ | $2.33$ | $\boldsymbol{4.53}$ | $6.76$ | $\boldsymbol{12.85}$ |
| Registrar 1 | $6.25$ | $11.74$ | $3.08$ | $\boldsymbol{6.23}$ | $4.25$ | $11.81$ | $9.64$ | $17.88$ |
| Registrar 2 | $5.86$ | $9.48$ | $4.66$ | $10.59$ | $6.4$ | $17.58$ | $19.89$ | $34.09$ |
| Registrar 3 | $4.48$ | $\boldsymbol{8.16}$ | $4.29$ | $8.23$ | $3.78$ | $6.98$ | $13.28$ | $14.27$ |
